# Patient engagement in decision making and associated factors among outpatients with selected non-communicable chronic diseases in public hospitals of West Shoa, Ethiopia

**DOI:** 10.1371/journal.pgph.0000772

**Published:** 2023-05-17

**Authors:** Desalegn Emana, Mulu Kitaba, Taka Girma, Shalama lekassa, Firaol Regea, Hunduma Dina, Segni Mulgeta

**Affiliations:** 1 Department of Nursing, College of Health Sciences, Assosa University, Assosa, Ethiopia; 2 Department of Nursing, College of Medicine and Health Sciences, Ambo University, Ambo, Ethiopia; 3 Department of Public Health, College of Medicine and Health Sciences, Ambo University, Ambo, Ethiopia; 4 Department of Public Health, College of Health Sciences, Assosa University, Assosa, Ethiopia; 5 Department of Midwifery, College of Health Sciences, Assosa University, Assosa, Ethiopia; 6 Department of Epidemiology, School of Public Health, Wollega University, Nekemte, Ethiopia; PLOS: Public Library of Science, UNITED STATES

## Abstract

**Background:**

Despite the importance of patient engagement in health care decision-making in the care of patients with chronic diseases, there is limited information about it and the factors affecting it in Ethiopia and in the Public Hospitals of West Shoa in particular. Thus, this study was designed to assess the engagement of patients with selected chronic non-communicable diseases in health care decision-making and associated factors in public hospitals of West Shoa Zone, Oromia, Ethiopia.

**Methods:**

We used an institution -based cross-sectional study design. We used systematic sampling for the selection of study participants from June 7–July 26, 2020. Standardized, pretested, and structured Patient Activation Measure was used to measure patient engagement in healthcare decision-making. We did descriptive analysis to determine the magnitude of patient engagement in health care decision-making. Multivariate logistic regression analysis was used to determine factors associated with patients’ engagement in the health care decision-making process. Adjusted odds ratio with a 95% confidence interval was calculated to measure the strength of association. We declared statistical significance at p<0.05. we presented the results in tables and graphs.

**Results:**

406 patients with chronic diseases took part in the study, yielding a response rate of 96.2%. Less than a fifth [19.5% (95% CI: 15.5, 23.6)] of participants in the study area had a high engagement in their health care decision-making. Educational level (college or above) [AOR = 5.2, 95% CI (1.76–15.46)], duration of diagnosis >5 years [AOR = 1.8, 95% CI (1.03–3.2)], health literacy [AOR = 1.15, 95% CI (1.06–1.24)], autonomy preference in decision making [AOR = 1.35, 95% CI (1.03–1.96)] were factors significantly associated with participants’ engagement in health care decision making among patients with chronic diseases.

**Conclusion:**

A high number of respondents had a low engagement in their health care decision-making. Preference for autonomy in decision making, educational level, health literacy, duration of diagnosis with the disease were factors associated with patient engagement in health care decision making among patients with chronic diseases in the study area. Thus, patients should be empowered to be involved in decision making to increase their engagement in the care.

## Introduction

Patient Health Engagement Model (PHE) defined patient engagement as, a multidimensional, psychosocial process that results from cognitive, emotional, and behavioral enacting of patients towards their disease and its management [1]. Further, it is defined as the degree of active involvement people have in taking care of their health and in determining their care [2]. Patient engagement is a dynamic and evolutionary process in which the patient proceeds through the four phases; blackout, arousal, adhesion, and eudemonic project, to achieve full engagement [1]. Across the phases, patients acquire knowledge and become more confident and differently engaged in decision making based on their emotional, cognitive and behavioral mindset [1, 3].

Chronic non-communicable diseases are the causes for two-thirds of deaths each year causing 42% of premature deaths,80% of which occurs in developing countries [4]. A recent study in Ethiopia showed that more than 75% of follow-up for chronic non-communicable disease is due to the three chronic diseases (heart diseases, hypertension, and diabetes mellitus) [5]. Another study in Ethiopia indicated that non-communicable chronic diseases were the leading causes of age-standardized death rate [6].

Non-communicable chronic diseases need lifelong care and need not only care providers; but also a paradigm shift to team-based care in which patient and care providers work together for control and prevention of disease-related complications [7]. This necessitates the involvement of patients in all aspects of the care, including decision-making around medical management [8, 9]. When patients engage in their healthcare decision-making, they are better able to make informed decisions about their care options, guarantee better use of resources, feel confident to put their viewpoints and promote mutual understanding and accountability with their health care providers. Moreover, it enhances patient and provider satisfaction [10], makes patients familiar with their medication or treatment plans, and finally increases adherence to their overall treatment [2, 11].

**Fig 1:**
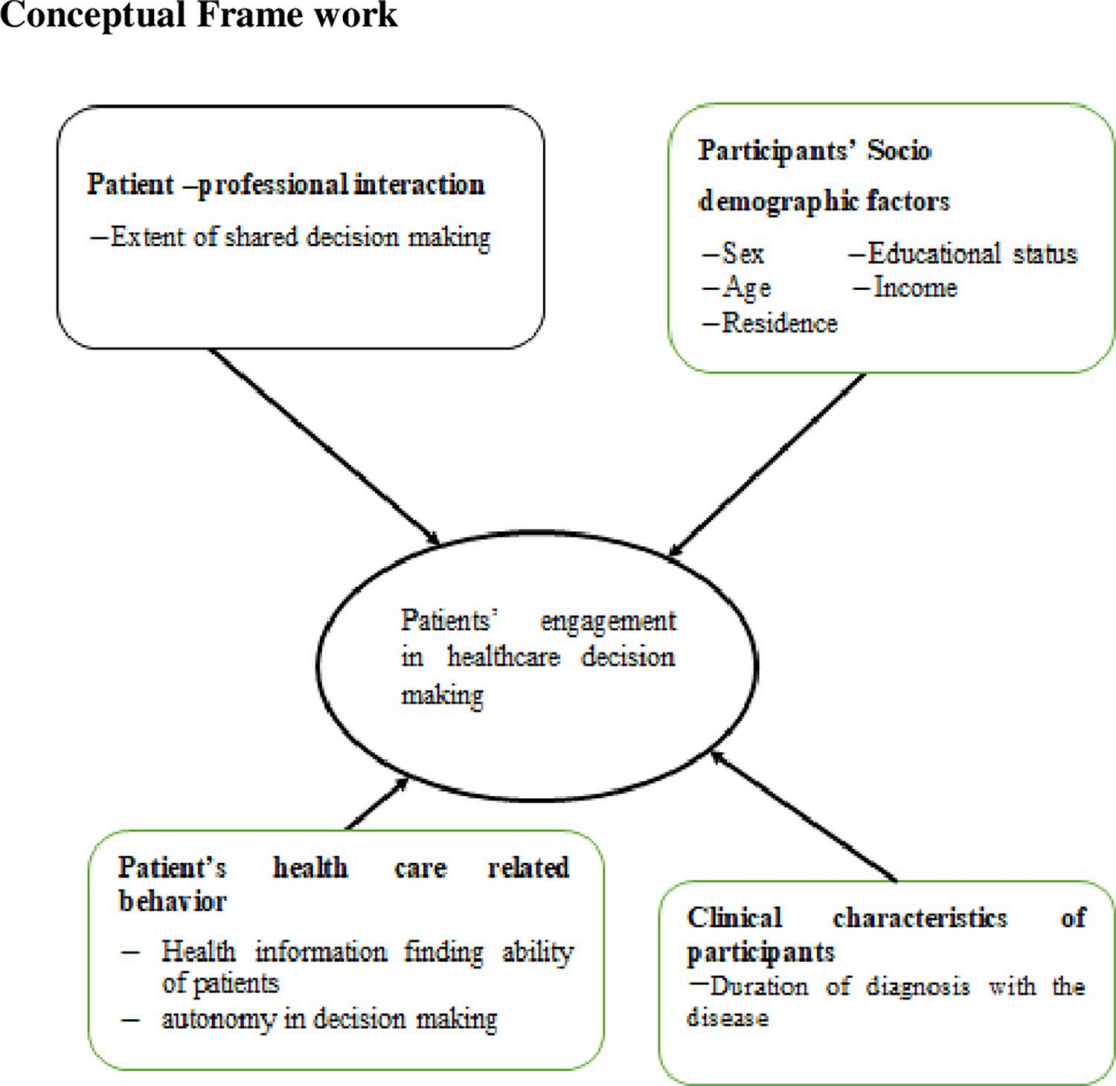
Conceptual framework on chronic patient’s engagement in health care decision making in public hospitals of West Shoa, central Ethiopia, 2020. Source: Adapted from review of literatures [16–23].

Chronic patients who have less active engagement levels are more likely to be hospitalized and re-admitted leading to higher health care costs [12]. Patients with lower engagement had health care expenses than those with higher engagement levels [13] and attend the emergency department frequently [14]. Low engagement in health care is also supposed to decrease implementation of chronic care thus increasing the burden of chronic diseases [15]. Less actively engaged individuals are more likely to have unmet medical needs and delay their medical care [2]. Further low engagement level is a risk for poor self-care behavior [16]. However, in our country, there is limited evidence on the status of patients’ engagement in decision-making in their health care and the factors affecting it. So the aim of this study is to point out the status of patients’ engagement in decision making as the issue was left aside in developing country in maintaining quality of care. Also it pinpoints some factors associated with patients’ engagement in decision making (Fig 1).

## Methods and materials

### Study area and period

The study was conducted in randomly selected public hospitals of west Shoa zone from June 7 –July 26, 2020. West Shoa is one of the zones of Oromia Regional State. It has 8 public hospitals. The hospitals were general hospitals. The service given in each hospitals were similar in scope. The hospitals have separate outpatient department for treatment of chronic diseases like cardiovascular diseases, diabetic mellitus, and other chronic diseases. This study aimed to be conducted in West Shoa as there were no previous studies indicating status of patient engagement in the study area. In addition, study area was selected for feasibility of the study.

#### Study design

Facility based cross sectional quantitative study design was used.

#### Source population

All patients with non-communicable chronic diseases who were 18 years old and above and who were on follow up at outpatient department in public Hospitals of West Shoa.

### Study population

All patients with non-communicable chronic diseases who were ≥18 years old and above who were on follow up at the randomly selected public hospitals of West Shoa Zone during study period.

### Eligibility criteria

#### Inclusion criteria

Patients with chronic non communicable diseases (cardiovascular diseases and Diabetes Mellitus) who were ≥18 years old and who have attended their appointment at least for 6 months; and those who were able to give response.

#### Exclusion criteria

Outpatients with selected chronic non-communicable diseases who were not able to respond to the interview.

### Sample size determination and sampling technique

The sample size for this study was calculated using single population proportion formula. It was calculated considering proportion (P) of patients’ engagement in health care decision making as 50%, margin of error (d) = 5% and confidence interval of 95%. Then final sample size was obtained by adding 10% non-response rate and it became 422.

#### Sampling technique

From eight hospitals in west Shoa, four Hospitals were randomly selected using simple random sampling. From the previous month’s patient flow, total of 936 chronic patients (597 patients with cardio vascular diseases and 339 patients with DM) were taken as base line for sampling. Then patients were proportionally allocated to each selected hospitals. Finally the required sample size was selected from each hospitals depending on the number of patients at each hospitals.

### Variables

#### Dependent variable

Patient engagement in health care decision making.

#### Independent variables

Socio-demographic characteristics of patients (Age, Sex, monthly income, place of residence, educational status), patients’ clinical characteristics (Duration of diagnosis with the disease), patients’ health related behaviors (Health literacy, Participants’ autonomy in decision making) were included.

### Operational definitions

#### High engagement in health care decision making process

Participants were said to have high engagement in health care decision making if scored equal and above 55.1% on patient activation measure.

#### Low engagement in health care decision making process

Participants were said have low engagement in health care decision making if scored below 55.1% on patient activation measure.

#### Shared decision making

Share decision making(SDM) is explained as an approach where health care providers and patients share the best existing indication when faced with the task of making decisions [24].

#### Health information finding ability

The degree to which individuals have the ability to find, understand, and use information and services to inform health-related decisions and actions for themselves and others.

#### Autonomy in decision making

The extent to which people prefer doctors or themselves to make specific management decisions.

### Data collection tools and technique

The standardized Patient Activation Measure(PAM-13) questionnaire was used to measure patient engagement in health care decision making process [25]. Autonomy Preference Index (API) was used to measure patients’ preference for autonomy in health care decision making [26]. Health literacy Questionnaire [27] was used to measure participants information finding ability.

### Data quality control and management

Prior to data collection, reliability of tools was checked on pretest. English language prepared questionnaires were translated to local language (Afaan Oromoo) and then back to English by different translators to check consistency of the translation. Further data quality was maintained through orientation of data collectors and supervisors on data collection tools.

### Data processing and analysis

Completed questionnaires were coded, entered and cleaned using EPIDATA version 3.1 computer program and then exported to SPSS version 23.0 for analysis. Descriptive statistics were used to calculate the frequency distribution, proportions (for categorical variables) and mean, standard deviation, range were used for continuous variables. The outcome variable was analyzed from the data collected using a PAM questionnaire by summing up the total engagement level from 13 items of the patient activation measure with 4 points Likert scale (1 = strongly disagree, 2 = disagree, 3 = Agree, 4 = strongly agree). It was treated as binary variable. Accordingly participants with score 55.1% or more were coded 1 = high engagement in decision making; and those with lower than 55.1% were coded 0 = low engagement in decision making. A Bi-variable logistic regression analysis was done to see the association between each independent variable and the outcome variables. Variables with p-value <0.25 in the bi-variable logistic regression analysis were a candidate for multivariable logistic regression analysis. The logistic regression model fitness was checked using Hosmer-Lemeshow. Multicollinearity was checked using VIF. Both crude and adjusted odds ratio along with 95% CI were estimated to measure the strength of association. The level of statistical significance was declared at a p-value of less than 0.05. Tables and figures were used to display the results.

### Ethical and legal consideration

An ethical clearance was obtained from Institutional Health Research Ethics Review Committee (IHRERC) of Ambo University College of Medicine and Health Science. Oral and written informed consent was taken from the participants by language they could understand(Afaan Oromoo) before the interview. Participants were given all-inclusive information about the study and were guaranteed of confidentiality and protection of their privacy before giving consent. The consent was read for participants because not all participants were literate and if they agree they sign before the interview. An information sheet was attached to first part of the questionnaire and then a participant’s signature was obtained and kept with each questionnaire. All the procedures were approved by the ethics review committees of Ambo University.

Participation in this study was fully voluntary. In addition, no personal identifiers were used on data collection questionnaire and the data obtained from the study participants were not accessed by anybody except the investigator and were kept confidentially. The participants were assured that they have the right to refuse or withdraw if they were not comfortable at any time. Further preventive measures against COVID-19 was taken throughout data collection period.

## Results

### Socio-demographic characteristics of participants

A total of 406 patients with chronic diseases were involved in the study making an overall response rate of 96.2%. Two hundred nineteen (51.5%) of the participants were males and majority (337(83.2%)) of the participants were above the age of 30 years (Table 1).

**Table 1:** Socio-demographic characteristics of patients with chronic diseases in public hospitals of West Shoa, Ethiopia (n = 406), 2020.

Variable	Categories	Frequency	Percentage
**Sex of participant**	Male	209	51.5
	Female	197	48.5
**Age of participant**	≤29	68	16.7
	30–44	118	29.1
	45–59	123	30.3
	≥60	97	23.9
**Religion**	Orthodox	151	37.2
	Protestant	185	45.6
	Muslim	33	8.1
	Wakefata	31	7.6
	Others*	6	1.5
**Residence place**	Urban	184	45.3
	Rural	222	54.7
**Ethnicity**	Oromo	368	90.64
	Amhara	31	7.64
	Gurage	7	1.72
**Marital status**	Single	74	18.2
	Married	284	70.0
	Widowed	35	8.6
	Divorced	13	3.2
**Educational status**	No formal education	90	22.2
	Elementary(grade 5–8)	169	41.6
	Secondary school(9–12)	85	20.9
	College/university	62	15.3
**Participant’s occupation**	Farmer	116	28.6
	Government employee	32	7.9
	Self-employee	144	35.4
	Retired	29	7.1
	Student	33	8.2
	House wife	52	12.8
**Participant’s average monthly income**	<1000ETB	316	77.8
	≥1000ETB	90	22.2
**Participants source of health information about their illness**	Reading book	30	7.4
	News paper	7	1.7
	Health care worker	406	100
	Television	97	23.6
	Radio	98	24.1
	Others**	8	2.0

* = Catholic, ‘Waaqa’ (‘Qaallicha’) ETB = Ethiopian birr

** = internet, friends, family members

### Participants’ clinical characteristics

Of the 406 patients with chronic diseases, 167(41.1%)) were hypertensive patients and 282 (69.5%) of the participants were less than 5 years since they started their follow up. Nearly a fifth of the participants (18.7%) had family history of the same chronic diseases and large proportions (70.9%) of the participants do not have history of previous hospitalization with their current disease (Table 2).

**Table 2:** Clinical characteristics of patients with chronic diseases in public hospitals of west Shoa, central Ethiopia (n = 406), July, 2020.

Variables	Categories	Number	Percentage
**Type of disease for which they are on follow up**	Hypertension	167	41.1
	Diabetes mellitus	144	35.5
	Heart disease	95	23.4
**Family history of the same chronic disease**	Yes	76	18.7
	No	330	81.3
**Duration since diagnosed with disease**	<5yrs.	282	69.5
	≥5 yrs.	124	30.5
**Previous history of hospitalization with the disease**	Yes	118	29.1
	No	288	70.9
**Patients’ awareness of their right to make treatment decision**	Yes	287	70.7
	No	119	29.3
**Do you search for information intentionally in advance to your health care provider’s advice?**	Yes	190	46.8
	No	216	53.2

### Participants’ preference for autonomy in decision making

The mean score and standard deviation of autonomy preference in decision making was 75.92 (SD ±11.6) with range 35 to 95. Majority of the participants(83.1%) agree that the important medical decision should be made by health care provider. On the other hand small percentage(12.8%) of participants disagree to go along with health care providers advice even if they disagree with it (Table 3).

**Table 3:** Preference for autonomy in decision making among patients with chronic diseases in public hospitals of West Shoa Zone, central Ethiopia 2020 (n = 406).

	Variables (items of preference for autonomy)	Disagree		Agree	
		F	%	F	%
**1**	The important medical decisions should be made by your care provider, not by you	69	16.9	337	83.1
**2**	You go along with your care provider’s advice even if you disagree with it.	52	12.8	354	87.2
**3**	You should not be making decisions about your own care	94	23.1	312	76.9
**4**	As your illness became worse you would want your doctor to take greater control	124	30.5	282	69.5

### Participants’ health information finding ability

The mean and standard deviation of health information finding ability score of participants was 10.48 (SD± 3.6). majority of the participants (82.26%) had difficulty in Finding information about their health problem. On the other hand only 33.5% of the participants can get easily up-to-date information about their illness (See Table 4).

**Table 4:** Participants’ health information finding ability in public hospitals of West Shoa Zone, central Ethiopia 2020 (n = 406).

Variables	Difficult		Easy	
	Frequency	percentage	Frequency	Percentage
**Find information about your health problem**	334	82.26	72	17.74
**Find health information from different places**	326	80.3	80	19.7
**Get information about your health so you are up to date about your illness**	270	66.5	136	33.5
**Get health information in words you understand**	279	68.7	127	31.3

### Patient engagement in healthcare decision making

**Fig 2:**
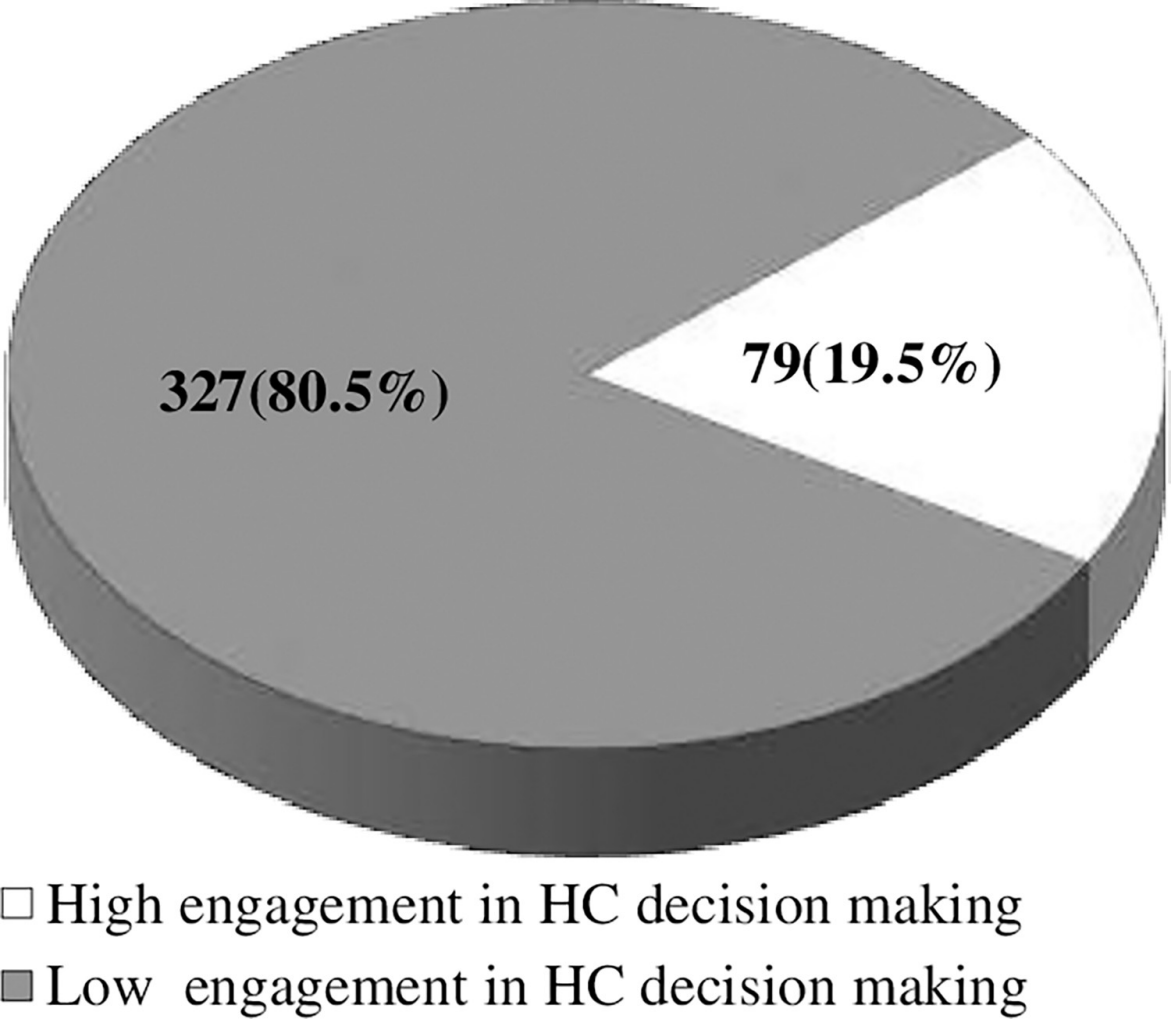
Patient engagement in decision making.

The mean score of participants’ engagement in health care decision making was 51.07 [(95% CI: 50.4, 51.7)] with standard deviation of (± 6.6) [(95% CI (5.9, 7.2)]. The minimum and maximum score of patients engagement in decision was 34.62 [CI(28.3–41.2)] and 75 [CI(69.7–78.5)] respectively. By dichotomizing the score, significantly lesser proportion; 19.5% [95% CI: 15.5, 23.6)] of the participants in the study had high engagement in their health care decision making process (Fig 2).

### Factors associated with patient engagement in health care decision making

In multivariable logistic regression analysis, participants with higher educational level (college or above) were about 5 times [AOR = 5.2, 95% CI (1.76–15.46)] more likely to be engaged in their health care decision making than those with no formal education. Participants with five year and above duration of diagnosis were about two times [AOR = 1.8, 95% CI (1.03–3.2)] more likely to be engaged in their health care decision making when compared with those who had duration of diagnosis less than five year.

The study also indicated, For every one point increase in participant’s health information finding ability, the log odds that the participants will have high engagement in their health care decision making increases by 0.14 [B = 0.14,AOR = 1.15,95% CI(1.06–1.24)] on average. On the other hand the log odds that participants will have high engagement in their health care decision making increases by 0.3[B = 0.3, AOR = 1.35, 95% CI (1.03–1.96)] on average for one point increase in participant’s preference for autonomy in decision making score at p-value less than 0.05 (Table 5).

**Table 5:** Factors associated with patient engagement in health care decision making (n = 406) in public hospital of West Shoa Zone, central Ethiopia, 2020.

variable	Categories	Patient engagement in health care decision making		Odds Ratio with 95% CI	
				Crude OR	Adjusted OR
		High	Low		
**Sex**	Male	37(9.1%)	172(42.4%)	1	1
	Female	42(10.3%)	155(38.2%)	1.26(0.77,2.06)	1.7(0.97,3.07)
**Age**	age ≤29	18(4.4%)	50(12.3%	2.3(1.03,5.1)*	1.2(0.46,3.13)
	age 30–44	29(7.1%)	88(21.7%)	2.1(1.02,4.32)*	1.18(0.5,2.8)
	age 45–59	19(4.7%)	106(26.1%)	1.14(0.53,2.4)	0.8(0.35,2.01)
	age ≥60	13(3.2%)	83(20.4%)	1	1
**Educational status**	No formal education	7(1.7%)	83(20.4%)	1	1
	Elementary grade	22(5.4%)	147(36.2%)	1.7(0.72,4.3)	2.1(0.82,5.42)
	Secondary school	20(4.9%)	65(16.0%)	3.6(1.45,9.15)*	3.4(1.26,9.6)**
	College/university	30(7.4%)	32(7.9%)	11.1(4.4,27.84)*	5.2(3.24,16.33)**
**Residence place**	Urban	44(10.8%)	140(34.5%)	1.67(1.02,2.75)*	1.1(0.62,1.95)
	Rural	35(8.6%)	187(46.1%)	1	1
**Monthly income**	<1000 ETB	50(12.3%)	266 (65.5%)	1	1
	≥1000 ETB	29(7.1%)	61(15.0%)	2.5(1.48,4.32)*	1.3(0.66,2.57)
**Duration of diagnosis**	≥5 years	36(8.9%)	88(21.7%)	2.27(1.37,3.77)*	1.8(1.03,3.2)**
	<5years	43(10.6%)	239 (58.9%)	1	1
**Preference for autonomy in**				1.54 (1,1.059)*	1.35(1.03,1.96)** β = 0.3
**Health literacy**				1.2(1.1,1.27)*	1.15(1.06,1.24)** β = 0.14

** Significant at p-value<0.05 ETB-Ethiopian Birr

OR = Odds ratio, CI = Confidence interval

## Discussion

This study was conducted to assess patient engagement in health care decision making and associated factors among patients with chronic diseases in public Hospitals of West Shoa Zone. It showed 19.5% of the participants had high engagement in their health care decision making and health literacy, preference for autonomy, educational level, duration of diagnosis were variables associated with patient engagement in health care decision making.

The finding of this study is lower than the finding of the study in Malaysia, Australia in which 61.8% and 68% of the participants respectively had high engagement [17, 28]. The reason for these disparities might be the difference in policy and health care system between the countries; like implementation of Teleprimary Care and Patient Centered Medical Home which is not in effect in our country. On the other hand the result is higher than the study finding in Nepal [29]; in which 6.6% of the of participants had high engagement. This difference might occur due to difference in the study population.

Participants with educational level at college or above were about five times (AOR = 5.2) more likely to engage in health care decision making compared with those who have no formal education. The reason might be more educated individuals are more involved in therapeutic communication than uneducated individuals and more committed to incorporate medical advice [30]. This result is supported by Study in Nepal [29], Turkey [20], and Australia [22].

Duration of diagnosis is significantly associated with patients’ engagement in health care decision making. Patients with duration of diagnosis greater than five years were about two times (AOR = 1.8) more likely to engage in their health care decision making when compared with their counter parts. This might be attributed to patient’s behavioral change overtime and experiential knowledge patients gained about their illness over a period of time [21]. The finding is in line with the study in Australia [22] and China [31]. But study in Ethiopia showed no significant association between duration of diagnosis and engagement in health care decision making [32].

The likelihood of engagement in decision making increase [(β = 0.14, AOR = 1.15)] as health information finding ability score increases. This might be due to individual’s initiation to ask questions and decide on sound treatment options as they build up knowledge about their medical conditions [33]. It is supported by Health Information Seeking Behavior Model and also in line with the findings of the study in Malaysia [28], Singapore [34], Denmark [35], Nepal [29] and England [36]. Contrary to this, study conducted in Canada revealed no association between patient engagement in healthcare decision making and health information finding ability [37]. The possible reason for this difference might goes to socio economic difference between the population; the difference might also goes to goes to small sample size of the study conducted in Canada, as sample size affects the association between variables.

Patients’ preference for autonomy was significantly associated (β = 0.3, AOR = 1.35) with their engagement in health care decision making. The reason behind this might be due to the self-determination to perform a specific action as autonomy increases and intrinsic motivation of individuals to make decision which is justified by Middle Range Theory of nursing and self-determination theory [38, 39]. Study finding from New Jersey [40] and Italy also support the present finding [23].

### Strength and limitations of the study

#### Strength

It was the only study in the study area to study the magnitude and factors affecting patient engagement.

#### Limitation

This study was not free of limitations. As the study design is cross-sectional, the design would not be able to draw a causal relationship between associated factors and patient engagement in decision making. Patients may not remember their exact duration of diagnosis as the duration gets longer; so that recall bias might be a problem.

#### Study implication

This study helps the government and policy makers in designing appropriate intervention toward patient engagement improvement. It also helps the health care providers to initiate patients to improve self-efficacy. The information obtained can also form a basis for other stakeholders and policy makers to plan on developing empowerment interventions to improve patients’ engagement in decision making in general. It also form basis for further studies on ‘why’ of the low patient engagement in health care decision making and will also be used as additional input for researchers in related topic and will elicit support and promote cooperation among different stakeholders towards the initiation of sustainable people centered health care program for patients with non-communicable chronic diseases or other similar circumstances in different parts of the country.

## Conclusion and recommendation

In conclusion, the result of this study showed that, participants’ engagement in their healthcare decision making is low and Health information finding ability, duration of diagnosis with the disease, educational level and patients’ preference for autonomy in decision making were variables significantly associated with patients’ engagement in health care decision making among patients with chronic diseases. Thus patient centered care and shared clinical decision making should be encouraged in the study area. In addition patient activation level needs to be measured at each follow up visit so as to check the improvement in patients’ engagement level.

## Supporting information

S1 Data SPSS data for analysis. (SAV)
